# New foe treated with old guns – supportive role of steroids in the treatment of acute severe hepatitis E

**DOI:** 10.1186/s12876-014-0191-0

**Published:** 2014-11-15

**Authors:** Marcial Sebode, Sven Pischke, Marc Lütgehetmann, Susanne Polywka, Alexander Quaas, Ansgar W Lohse, Henning Wege

**Affiliations:** I. Department of Internal Medicine, University Medical Center Hamburg-Eppendorf, Martinistr. 52, 20246 Hamburg, Germany; Outpatient Clinic for Liver Transplantation, University Medical Center Hamburg-Eppendorf, Hamburg, Germany; Department of Medical Microbiology, Virology and Hygiene, University Medical Center Hamburg-Eppendorf, Hamburg, Germany; Department of Pathology, University Medical Center Hamburg-Eppendorf, Hamburg, Germany

**Keywords:** Hepatitis E, Autochthonous, Acute liver injury, Steroid treatment

## Abstract

**Background:**

Autochthonous hepatitis E has been observed with growing incidence in industrialized countries. Hepatitis E virus infection causes an acute hepatitis with spontaneous resolution in the majority of cases. However, in individual cases, hepatitis E may lead to life-threatening acute liver failure. In this report, we describe a case of acute liver injury caused by an autochthonous hepatitis E that resolved under steroid treatment. To our knowledge, this is the first case report describing supportive steroid monotherapy for acute liver injury due to hepatitis E.

**Case presentation:**

A 63-year-old Caucasian male presented with acute liver injury of unknown origin. After excluding the most prevalent causes of acute liver injury, liver histology revealed signs of immune-mediated toxic or drug-induced liver injury. Therefore, immunosuppressive treatment with prednisolone was started. After initialization of steroid treatment, polymerase chain reaction analyses of peripheral blood and liver tissue revealed an acute hepatitis E virus infection (genotype 3). Under sustained steroid treatment, acute liver injury improved and hepatitis E infection resolved.

**Conclusion:**

Steroid treatment might be an option to prevent progress of life-threatening liver failure and liver transplantation in patients with hepatitis E-induced acute liver injury and high-grade inflammation.

## Background

Hepatitis E (HEV) is a single-stranded RNA-virus and its genome comprises three overlapping open reading frames (ORF). Four human pathogenic HEV genotypes are represented by one serotype. Autochthonous hepatitis E in industrialized countries is due to HEV genotype 3 and 4. These genotypes infect both humans and animals, such as pigs, rodents and shellfish [1]. Risk factors for clinically apparent autochthonous HEV infection are male sex and older age [2]. Chronic alcohol consumption additionally seems to promote a more severe course of HEV infection [3]. An important differential diagnosis for hepatitis E is drug-induced liver injury (DILI). It has been shown that acute autochthonous HEV has been frequently misdiagnosed as DILI in the past [4], thus the awareness for the diagnosis of hepatitis E needs to be increased.

The diagnosis of acute HEV infection is based on the detection of HEV RNA by polymerase chain reaction (PCR) and/or anti-HEV IgM. Anti-HEV IgG emerges normally in the later course of resolving hepatitis E. Immunoassays vary in their accuracy and are not reliable for making a proper diagnosis of acute HEV infection [5]. Thus, confirmation of hepatitis E should rely on highly sensitive HEV-RNA PCR out of blood or stool samples [1]. However, a recent investigation showed a variable sensitivity for different HEV PCR assays [6]. False negative test results may lead to undiagnosed cases with possible severe complications [7]. As of now, no test for detection of HEV has been approved for clinical use in the USA by the U.S. Food and Drug Administration.

Hepatitis E can induce liver failure and, in consequence, liver transplantation may be required. Most of these fulminant HEV infections are due to endemic genotype 1, especially in pregnant women and patients with chronic liver disease [8,9]. In autochthonous HEV infections caused by genotype 3, acute hepatitis usually resolves spontaneously within 4–6 weeks [10]. However, hepatic failure can occur as a rare complication [3]. In case series of hepatic failure by hepatitis E genotype 1 and 3, ribavirin has successfully been used to prevent liver transplantation [11,12].

We here report a case of acute liver injury caused by an autochthonous HEV genotype 3 infection that resolved under steroid treatment.

## Case presentation

A 63-year-old male was referred to our liver unit because of acute liver injury of unknown origin. One day before admission, he presented himself at the emergency room with a rapid onset and painless jaundice. In the week before, he had had two episodes of chills and night sweats and observed clinical signs of cholestasis, in particular dark urine and pale stools. In the past medical history, the patient suffered from high blood pressure, an asymptomatic cholecystolithiasis and colonic diverticulosis. His only medication was 10 mg of bisoprolol per day for many years. No allergies and no herbal or over-the-counter remedies were reported. He regularly drank 0.5 liters of beer per day. He did not smoke or use illicit drugs. There was no history of surgery or blood transfusions. Consumption or contact to game meat in the last 6 months was also excluded. He was married and had no changing sexual partners. In his family, no autoimmune or genetic diseases were known. He was a retired truck driver and bred poultry as a hobby.

On clinical examination, the patient was in an obese condition with a body-weight of 100 kg. He presented with jaundice. No clinical signs of liver cirrhosis were detected. The abdomen was slightly tender in the right upper quadrant and the liver was extended 4 to 5 cm below the costal margin. An enlargement of the spleen was not detected. There were no clinical signs of hepatic encephalopathy.

On admission, laboratory results revealed an acute liver injury with high transaminases (Table 1). Additional laboratory results on admission were CRP (C reactive protein) 26 mg/l (normal range <5 mg/l), antithrombin III 51.8% (70-130%), ferritin 22093.0 μg/l (34–400 μg/l), transferrin saturation 95% (16-45%), ceruloplasmin 0.30 g/l (0.22-0,61 g/l) and ANA (antinuclear antibodies) 1:80. AMA (antimitochondrial antibodies), SMA (smooth muscle antibodies), LKM (liver-kidney-microsome antibodes) and SLA/LP-(soluble liver antigen/liver pancreas antigen) antibodies were negative. We excluded hepatotropic viral infections, especially hepatitis A, B and C virus, as well as cytomegalovirus, Epstein-Barr-virus, adenoviruses and herpes simplex virus. On the first day in our unit, an immunoblot for anti-HEV IgG was intermediate positive. Anti-HEV IgM could not be detected. An initial commercial qualitative PCR (Genosis, Primerdesign, Southhampton, UK) was negative for HEV-RNA.

**Table 1:** **Laboratory data**

**Variable**	**Reference range**	**On admission**	**2nd day (starting steroid treatment)**	**After 1 week**	**After 3 weeks**
Hemoglobin (g/dl)	14.0-17.5	15.0	13.7	14.4	14.2
White-cell count	3.8-11.0	4.6	3.4	7.2	10.6
(per mm^3^)					
Platelet count	150-400	113	111	226	128
(per mm^3^)					
International normalized ratio		2.01	2.14	1.67	1.17
Urea nitrogen (mg/dl)	8-26	13	9	11	16
Creatinine (mg/dl)	0.6-1.3	0.8	0.9	1.0	1.2
Bilirubin total (mg/dl)	<1.2	7.7	8.7	9.4	4.4
Alkaline phosphatase (U/l)	40-129	154	125	139	161
Gamma-glutamyl transpeptidase (U/l)	<65	312	242	391	345
Alanine aminotransferase (U/l)	10-50	6635	4963	1331	200
Aspartate aminotransferase (U/l)	10-50	5133	2950	96	55
Lactate dehydrogenase (U/l)	87-241	1144	592	243	201
Albumin (g/l)	35-50	33	26	26	30

**Figure 1:**
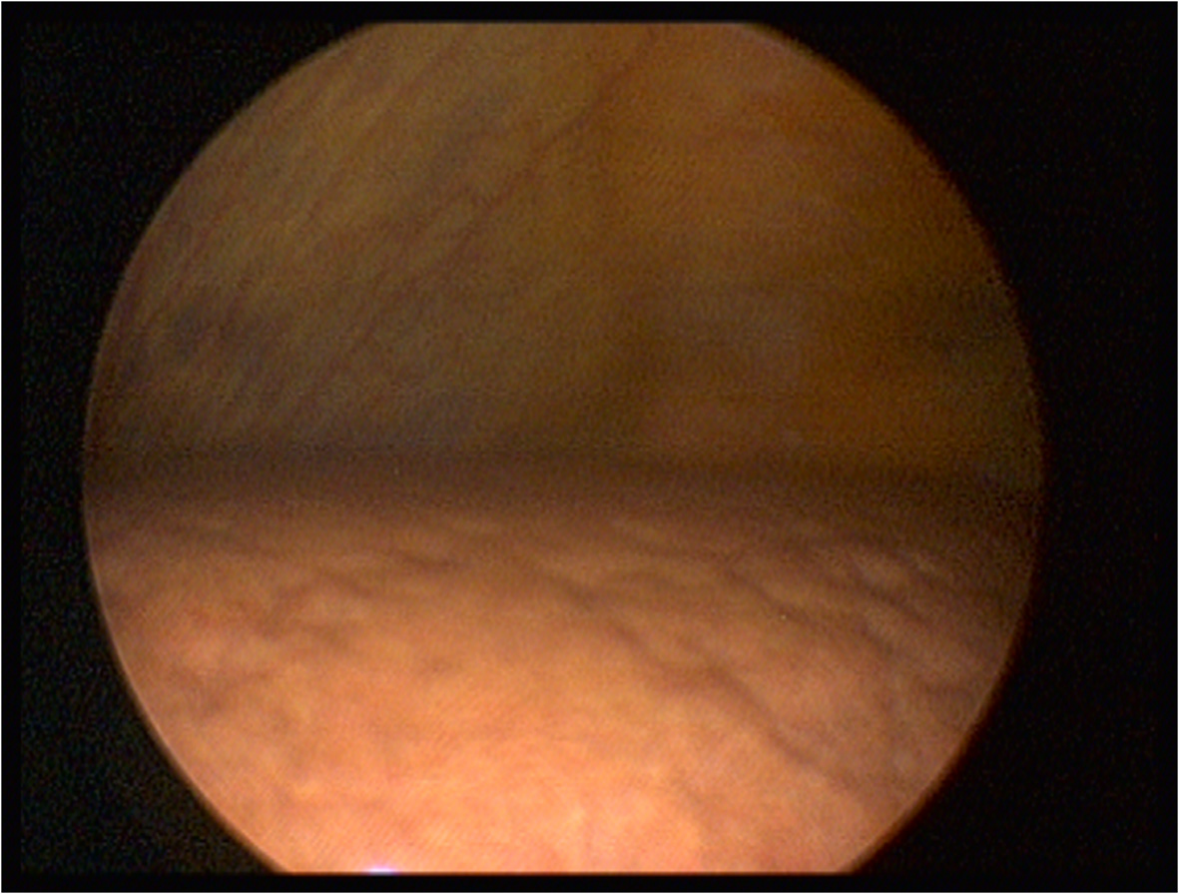
**Laparoscopic liver examination.** Retractions of the liver surface indicate focal necrosis, which is typical for acute liver injury.

**Figure 2:**
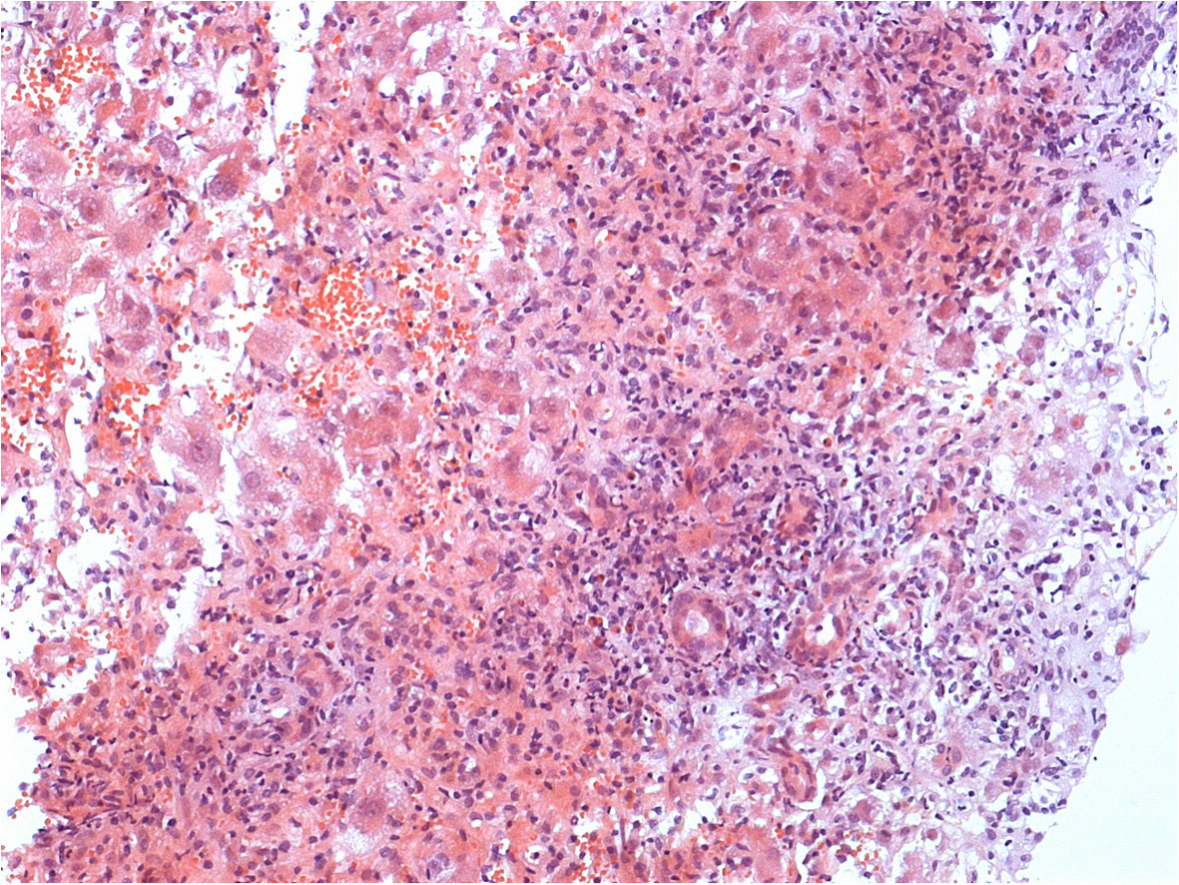
**Liver histology of acute hepatitis E.** Acute lobular hepatitis with signs of regeneration; cholestatic hepatitis was also present, in this section represented by proliferating neoductuli.

Abdominal ultrasound showed no signs of liver cirrhosis or intrahepatic masses. Ascites was absent. The gall bladder and the biliary tract were regular. A vascular pathology, such as portal or liver vein thrombosis, was ruled out. On the day after admission, we performed a laparoscopy to examine the liver and to obtain a liver biopsy. On laparoscopic liver examination, retractions of the liver surface could be detected as indication for focal necrosis, typical for acute liver injury (Figure 1). There was no evidence of chronic liver injury or cirrhosis. On histopathology, an acute lobular and cholestatic hepatitis was revealed (Figure 2). There were no typical histological signs of iron overload and steatohepatitis. Based on liver histology, an immune-mediated toxic or drug-induced hepatitis was favored.

The initial working diagnosis was an allergic drug-induced acute liver injury caused by an unidentified substance. Based on distinct immune cell infiltrates in liver biopsy and high risk of acute liver failure, an immunosuppressive therapy with prednisolone (1 mg per kilogram body weight per day) was started immediately after liver biopsy on the second day in our unit. Following chemical response, prednisolone was reduced in a stepwise fashion by 10 mg per week down to a daily dose of 20 mg and then reduced in 5 mg steps per week.

**Figure 3:**
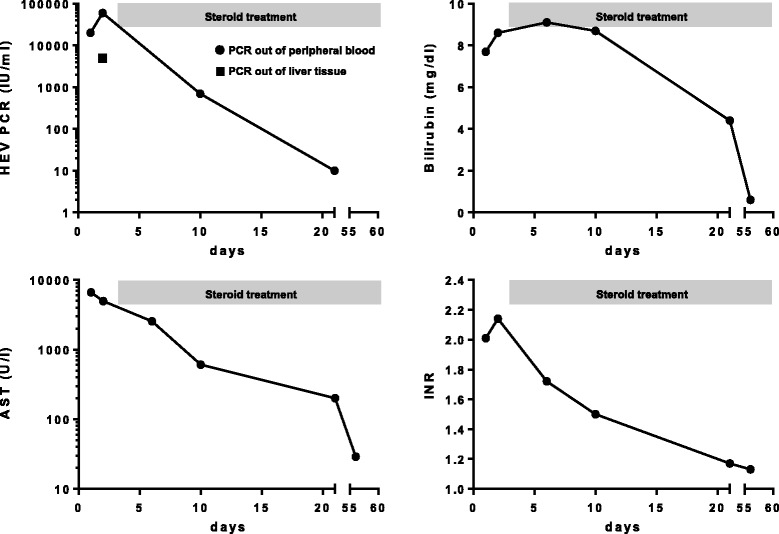
**Laboratory data and HEV PCR results after and before steroid treatment.** Course of laboratory data and HEV PCR before and after starting immunosuppressive treatment; grey bars represent duration of steroid treatment; prednisolone was started with 1 mg per kilogram bodyweight per day and reduced in a stepwise fashion.

Despite negative initial qualitative PCR analysis for HEV ORF2, we suspected acute HEV infection: The patient had risk factors for acute HEV infection (age, sex, clinical presentation) and an agent for DILI could not be identified in repeated interviews. Therefore and because anti-HEV IgG was intermediate positive, PCR for HEV was repeated by two different real time PCR assays based on the detection of ORF3 HEV (Altona Diagnostics, Hamburg Germany) and a quantitative in-house PCR [13]. Finally, both quantitative PCR analyses were positive and HEV genotyping confirmed HEV genotype 3 infection as the reason for acute liver injury in this patient. As additional confirmation, HEV-RNA was also detected in the liver tissue by both quantitative PCR assays based on ORF3 target region. For viral quantification, the new WHO standard was used and results were normalized to IU/ml. Under sustained steroid treatment, hepatitis E resolved adequately with rapidly declining viral load (Figure 3). Hepatic encephalopathy did not emerge in the further clinical course. The ALT level declined to 50% of its initial value three days after starting steroids. Besides, a 50% reduction of bilirubin was observed 15 days after beginning the steroid course. Because of the benign course under sustained treatment, we decided to continue steroids and did not add ribavirin, which would have been an option for acute severe HEV infection [11,12].

## Conclusions

In this case, diagnosis of acute HEV infection was delayed and acute liver injury was initially diagnosed as related to an immune-mediated toxic or drug-induced liver injury. Initial ELISA assays showed only an intermediate positive reaction for anti-HEV IgG and a negative reaction for anti-HEV IgM. The latter is probably due to a false negative result and underlines the need for PCR analyses for making an accurate diagnosis of acute HEV infection. Since the initial PCR analysis was also negative for HEV, the final diagnosis needed the use of an alternative PCR assay in this case. Interestingly, a recent study indicated that assays which detect a region in the ORF2-3 junction are more sensitive and robust than assays relying on ORF2, probably since this region seems to be less conserved between the HEV genotypes [14].

Because of the fulminant course with progressive acute liver injury and the immune cell infiltrate observed in the liver biopsy specimen, immunosuppressive treatment with prednisolone was started as an emergency treatment to decelerate liver injury caused by ongoing hepatitis. At the time the diagnosis of HEV was established, we decided to continue steroid treatment because transaminases declined, liver function restored, and viral load resolved.

Immunosuppressive treatment for acute viral hepatitis E seems to be counterproductive and even contraindicated at first glance. Compromising the immune response would entail the risk of restraining viral clearance or may even promote a chronic course of infection. Indeed, chronic autochthonous HEV infection has been mainly described for immunocompromised patients, particularly after solid-organ-transplantation, in patients with haematological malignancies or co-infection with human immunodeficiency virus [15]. In addition, chronic hepatitis E has also been described in patients with immune-mediated diseases, for example idiopathic CD4-disturbance or systemic lupus erythematodes under immunosuppressive or immune-modulating treatment [16,17]. However, the present case demonstrates that short-term steroid medication might be beneficial in HEV-induced acute liver injury, especially considering that even in HEV infection acute liver injury is likely immune-mediated and not due to the viral replication per se [18]. Along this line, recent data point at a non-cytopathic, immune-mediated hepatocyte damage caused by HEV [19,20]. Further investigations are certainly necessary to get insight into immune mechanisms caused by HEV in the acute and chronic disease to deliver adequate therapy for patients.

A full literature research revealed another case report describing recovery from HEV infection under immunosuppressive treatment [21]. In that case, HEV was misdiagnosed and treated like acute autoimmune hepatitis. Taken together, immunosuppressive treatment of the inflammatory flare of acute HEV infection most likely does not hinder viral clearance, but may help to avoid additional liver damage and avert the need for liver transplantation.

It would be beneficial to identify patients with HEV-induced acute liver injury who might profit from immunosuppression and those who are likely to resolve HEV infection spontaneously without risk for acute liver failure. As major limitation for the interpretation of our case, the favourable outcome might just reflect the natural course of HEV infection that might have developed even without steroid treatment. Our patient presented with major risk factors (regular alcohol consumption and older age) for a severe course of HEV, which were identified by Péron et al. [3]. It is tempting to assume that steroid treatment prevented progression to encephalopathy. Still, we cannot rule out that absence of hepatic encephalopathy reflects the benign natural clinical course in this patient and is not a result of steroid treatment. If autochthonous HEV infection becomes fulminant, high-dose steroids might be a supportive treatment, potentially even additive to ribavirin that has already been used successfully in some case reports [11,12]. Ribavirin has also been applied as treatment of acute autochthonous HEV infection on pre-existing chronic liver disease [22].

This case highlights the possibility of steroid treatment as a therapeutic rescue in patients with hepatitis E. Further studies are required to evaluate whether steroid treatment has a supportive role for patients with HEV-induced acute liver injury and improves their spontaneous outcome. At least, steroid treatment does not seem to impair viral clearance. Therefore, steroid use should not be delayed in severe acute immune-mediated hepatitis if HEV infection is assumed and diagnostic test results are pending.

## Consent

Written informed consent was obtained from the patient for publication of this case report and any accompanying images. A copy of the written consent is available for review by the editor of this journal.

## Abbreviations

ANA: Antinuclear antibodies
AMA: Antimitochondrial antibodies
AST: Alanine aminotransferase
CRP: C reactive protein
DILI: Drug-induced liver injury
HEV: Hepatitis e virus
Ig: Immunoglobulin
LKM: Liver-kidney-microsome
PCR: Polymerase chain reaction
ORF: Open reading frame
RNA: Ribonucleic Acid
SLA/LP: Soluble liver antigen/liver pancreas antigen
SMA: Smooth muscle antibodies
